# Deep Learning-Based Approaches for Decoding Motor Intent From Peripheral Nerve Signals

**DOI:** 10.3389/fnins.2021.667907

**Published:** 2021-06-23

**Authors:** Diu K. Luu, Anh T. Nguyen, Ming Jiang, Jian Xu, Markus W. Drealan, Jonathan Cheng, Edward W. Keefer, Qi Zhao, Zhi Yang

**Affiliations:** ^1^Department of Biomedical Engineering, University of Minnesota, Minneapolis, MN, United States; ^2^Fasikl Incorporated, Minneapolis, MN, United States; ^3^Department of Computer Science and Engineering, University of Minnesota, Minneapolis, MN, United States; ^4^Department of Plastic Surgery, University of Texas Southwestern Medical Center, Dallas, TX, United States; ^5^Nerves Incorporated, Dallas, TX, United States

**Keywords:** convolutional neural network, deep learning, feature extraction, motor decoding, neuroprosthesis, neural decoder, peripheral nerve interface, recurrent neural network

## Abstract

Previous literature shows that deep learning is an effective tool to decode the motor intent from neural signals obtained from different parts of the nervous system. However, deep neural networks are often computationally complex and not feasible to work in real-time. Here we investigate different approaches' advantages and disadvantages to enhance the deep learning-based motor decoding paradigm's efficiency and inform its future implementation in real-time. Our data are recorded from the amputee's residual peripheral nerves. While the primary analysis is offline, the nerve data is cut using a sliding window to create a “pseudo-online” dataset that resembles the conditions in a real-time paradigm. First, a comprehensive collection of feature extraction techniques is applied to reduce the input data dimensionality, which later helps substantially lower the motor decoder's complexity, making it feasible for translation to a real-time paradigm. Next, we investigate two different strategies for deploying deep learning models: a one-step (1S) approach when big input data are available and a two-step (2S) when input data are limited. This research predicts five individual finger movements and four combinations of the fingers. The 1S approach using a recurrent neural network (RNN) to concurrently predict all fingers' trajectories generally gives better prediction results than all the machine learning algorithms that do the same task. This result reaffirms that deep learning is more advantageous than classic machine learning methods for handling a large dataset. However, when training on a smaller input data set in the 2S approach, which includes a classification stage to identify active fingers before predicting their trajectories, machine learning techniques offer a simpler implementation while ensuring comparably good decoding outcomes to the deep learning ones. In the classification step, either machine learning or deep learning models achieve the accuracy and F1 score of 0.99. Thanks to the classification step, in the regression step, both types of models result in a comparable mean squared error (MSE) and variance accounted for (VAF) scores as those of the 1S approach. Our study outlines the trade-offs to inform the future implementation of real-time, low-latency, and high accuracy deep learning-based motor decoder for clinical applications.

## 1. Introduction

Upper-limb amputation affects the quality of life and well-being of millions of people in the United States, with hundreds of thousand new cases annually (Ziegler-Graham, 2008). Neuroprosthetic systems promise the ultimate solution by developing human-machine interfaces (HMI) that could allow amputees to control robotic limbs using their thoughts (Harris, 2011; Johannes, 2011; Schultz, 2011; Cordella, 2016). It is achieved by decoding the subject's motor intent with neural data acquired from different parts of the nervous system. Proven approaches include surface electromyogram (EMG) (Sebelius, 2005; Fougner, 2012; Jiang, 2012; Amsuss, 2013; Zuleta, 2019; George, 2020a,b), electroencephalogram (EEG) (Hu, 2015; Zeng, 2015; Sakhavi, 2018; Kwon, 2019), cortical recordings (Mollazadeh, 2011; Hochberg, 2012; Irwin, 2017), and peripheral nerve recordings (Micera, 2011; Davis, 2016; Vu, 2017, 2020; Wendelken, 2017; Zhang, 2017; Nguyen and Xu, 2020).

However, implementing an effective HMI for neuroprostheses remains a challenging task. The decoder should be able to predict the subject's motor intents accurately and satisfy certain criteria to make it practical and useful in daily lives (Vujaklija, 2017; Krasoulis, 2019). Some criteria include *dexterity*, i.e., controlling multiple degrees-of-freedom (DOF) such as individual fingers; *intuitiveness*, i.e., reflecting the true motor intent in mind, and *real-time*, i.e., having minimal latency from thoughts to movements.

In recent years, deep learning techniques have emerged as strong candidates to overcome this challenge thanks to their ability to process and analyze biological big data (Mahmud, 2018). Our previous work (Nguyen and Xu, 2020) shows that neural decoders based on the convolutional neural network (CNN) and recurrent neural network (RNN) architecture outperform other “classic” machine learning counterparts in decoding motor intents from peripheral nerve data obtained with an implantable bioelectric neural interface. The deep learning-based motor decoders can regress the intended motion of 15 degrees-of-freedom (DOF) simultaneously, including flexion/extension and abduction/adduction of individual fingers state-of-the-art performance metrics, thus complying with the dexterity and intuitiveness criteria.

Here we build upon the foundation of Nguyen and Xu (2020) by exploring different strategies to optimize the motor decoding paradigm's efficiency. The aim is to lower the neural decoder's computational complexity while retaining high accuracy predictions to make it feasible to translate the motor decoding paradigm to real-time operation suitable for clinical applications, especially when deploying in a portable platform. This paper does not aim to solve the real-time problem but to study different approaches, highlighting their advantages and disadvantages, which will inform future implementation of the motor decoding paradigm in real-time.

First, we utilize feature extraction to reduce data dimensionality. By examining the data spectrogram, we learn that most of the signals' power concentrates in the frequency band 25–600 Hz (Nguyen and Xu, 2020). Many feature extraction techniques (Zardoshti-Kermani, 1995; Phinyomark, 2009, 2012; Rafiee, 2011) have been developed to handle signals with similar characteristics. Feature extraction aims not only to amplify the crucial information, lessen the noise but also to substantially reduce the data dimensionality before feeding them to deep learning models. This could simultaneously enhance the prediction accuracy and lower the deep learning models' complexity. Here we focus on a comprehensive list of 14 features that consistently appear in the field of neuroprosthesis.

Second, we explore two different strategies for deploying deep learning models: the two-step (2S) and the one-step (1S) approaches. The 2S approach consists of a classification stage to identify the active fingers and a regression stage to predict the trajectories of digits in motion. The 1S approach only has one regression stage to predict the trajectories of all fingers concurrently. In practice, the 2S approach should be marginally more efficient because not all models are inferred at a given moment. The models in the 2S approach are only trained on the subset where a particular finger is active, while all models in the 1S approach are trained on the full dataset. Here we focus on exploring the trade-offs between two approaches to inform future decisions of implementing the deep learning-based motor decoder in real-world applications.

The rest of this paper is organized as follows: Section “Data Description” introduces the human participant of this research, the process of collecting input neural signals from the residual peripheral nerves of the participant, and establishing the ground-truth for the motor decoding paradigm using deep learning models. Section “Data Preprocessing” elaborates on how to cut raw input neural data into trials and extract their main features in the temporal domain before feeding to deep learning decoding models. Section “Proposed Deep Learning Models and Decoding Strategies” discusses the two approaches to efficiently translate motor intent from the residual peripheral nerves of the participant into motor control of the prosthesis as well as the architecture and the hyper-parameters of the deep learning models used in each approach. Section “Experimental Setup” is about the three machine learning models used as the baseline and how input neural data are allocated to the training and validation set. Section “Metrics and Results” presents the metrics to measure the performance of all models used in the motor intent decoding process and discusses the main results of both proposed approaches. Section “Discussion” discusses the role of feature extraction in reducing the deep learning motor decoders' complexity for real-time applications, how to further apply it in future works, and the advantages of machine learning and deep learning motor decoders in different scenarios where input dataset's size varies. Finally, section “Conclusion” summarizes the main contributions of this paper.

## 2. Data Description

### 2.1. Human Participant

The human experiment is a part of the clinical trial DExterous Hand Control Through Fascicular Targeting (DEFT), which is sponsored by the DARPA Biological Technologies Office as part of the Hand Proprioception and Touch Interfaces (HAPTIX) program, identifier No. NCT02994160^1^. The human experiment protocols are reviewed and approved by the Institutional Review Board (IRB) at the University of Minnesota (UMN) and the University of Texas Southwestern Medical Center (UTSW). The amputee voluntarily participates in our study and is informed of the methods, aims, benefits, and potential risks of the experiments prior to signing the Informed Consent. Patient safety and data privacy are overseen by the Data and Safety Monitoring Committee (DSMC) at UTSW. The implantation, initial testing, and post-operative care are performed at UTSW by Dr. Cheng and Dr. Keefer, while motor decoding experiments are performed at UMN by Dr. Yang's lab. The clinical team travels with the patient in each experiment session. The patient also completes the Publicity Agreements where he agrees to be publicly identified, including showing his face.

**Figure 1:**
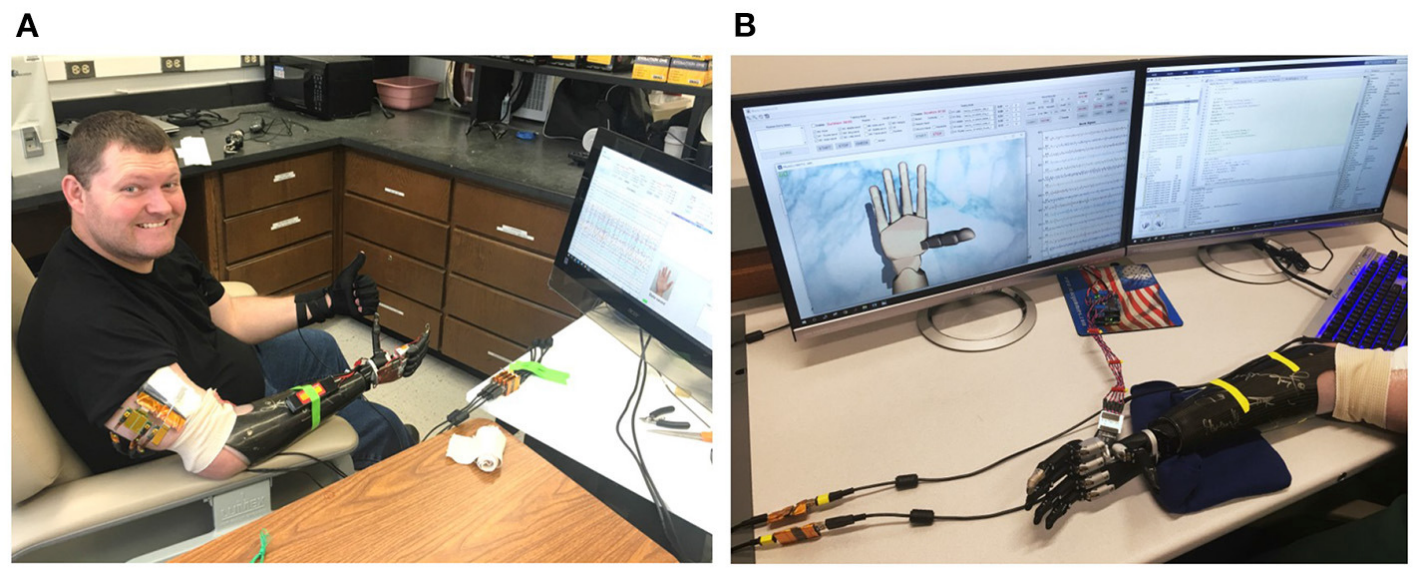
Photo of the **(A)** amputee and **(B)** the data collection software during a training session. The patient performs various hand movements repeatedly during the training session. Nerve data and ground-truth movements are collected by a computer and displayed in real-time on the monitor for comparison.

The participant is a transradial male amputee who has lost his hand for over 5 years (Figure 1). Among seven levels of upper-limb amputation, transradial is the most common type that accounts for about 57% of upper-limb loss in the U.S. (Schultz, 2011; Cordella, 2016). Like most amputees, the subject still has phantom limb movements; however, such phantom feelings fade away over time. By successfully decoding neural signals from the residual nerves of an amputee who has lost his limb for a long time, we would offer a chance to regain upper-limb motor control for those who are sharing the same conditions.

**Figure 2:**
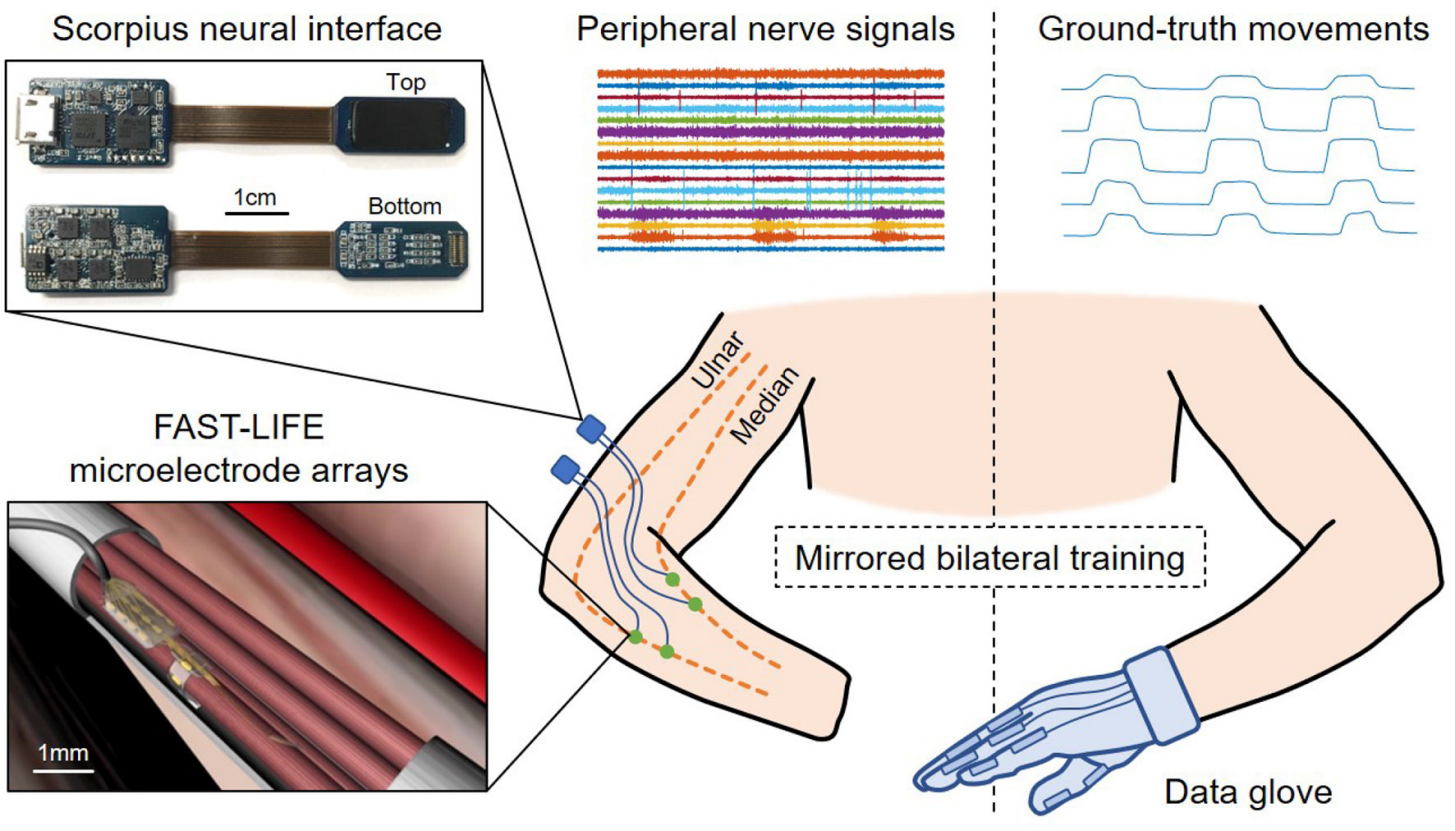
Overview of the human experiment setup and data acquisition using the mirrored bilateral training. The patient has four FAST-LIFE microelectrode arrays implanted in the residual ulnar and median nerve (Overstreet, 2019). Peripheral nerve signals are acquired by two Scorpius neural interface devices (Nguyen and Xu, 2020). The ground-truth movements are obtained with a data glove.

The patient undergoes an implant surgery where four longitudinal intrafascicular electrode (LIFE) arrays are inserted into the residual median and ulnar nerves using the microsurgical fascicular targeting (FAST) technique (Figure 2). The electrode array' design, characteristics, and surgical procedures are reported in Cheng (2017) and Overstreet (2019). The patient has the electrode arrays implanted for 12 months, during which the conditions of the implantation site is regularly monitored for signs of degradation.

The patient participates in several neural stimulations, neural recording, and motor decoding experiment sessions. He initially has weak phantom limb movements due to reduced motor control signals in the residual nerves throughout the years. However, the patient reports that the more experiment sessions he takes part in, the stronger his phantom control and sensation of the lost hand become. This suggests that training may help re-establish the connection between the motor cortex and the residual nerves, resulting in better motor control signals.

### 2.2. Nerve Data Acquisition

Nerve signals are acquired using the Scorpius neural interface (Figure 2)–a miniaturized, high-performance neural recording system developed by Yang's lab at UMN. The system employs the Neuronix chip family, which consists of fully-integrated neural recorders designed based on the frequency shaping (FS) architecture (Xu, 2014, 2020; Yang, 2016, 2018, 2020). The specifications of the Scorpius system are reported in Nguyen and Xu (2020). The system allows acquiring nerve signals with high-fidelity while suppressing artifacts and interference. Here two Scorpius devices are used to acquire signals from 16 channels across four microelectrode arrays at a sampling rate of 40 kHz (7.68 Mbps, 480 kbps per channel). The data are further downsampled to 5 kHz before applying a bandpass filter in 25–600 Hz bandwidth to capture most of the signals' power. This results in a pre-processed data stream of 1.28 Mbps (80 kbps per channel).

### 2.3. Ground-Truth Collection

The mirrored bilateral training paradigm (Sebelius, 2005; Jiang, 2012) is used to establish the ground-truth labels needed for supervised learning (Figure 2). The patient performs various hand gestures with the able hand while simultaneously imagining doing the same movement with the phantom/injured hand. During the ground-truth collection, the virtual hand and/or motorized prosthesis hand can follow the glove's movement to provide additional visual cues for the amputee. The ground truth is recorded while the patient poses his arms in different positions, including holding arms overhead, spreading to two sides, reaching the front, and resting along the body. The gestures include bending the thumb, index, middle, ring, little finger, index pinch, tripod pinch, grasp/fist, and resting. We record the training data in multiple sessions. During each session, the amputee performs one hand gesture 100 times, each time 4 s altering between resting and flexing. Peripheral nerve signals are acquired from the injured hand with the Scorpius system, while ground-truth movements are captured with a data glove (VMG, 30, Virtual Motion Labs, TX) from the able hand. The glove can acquire up to 15 DOF; however, we only focus on the main 10 DOF (MCP and PIP) corresponding to the flexion/extension of five fingers.

## 3. Data Preprocessing

### 3.1. Cutting Raw Neural Data

**Figure 3:**
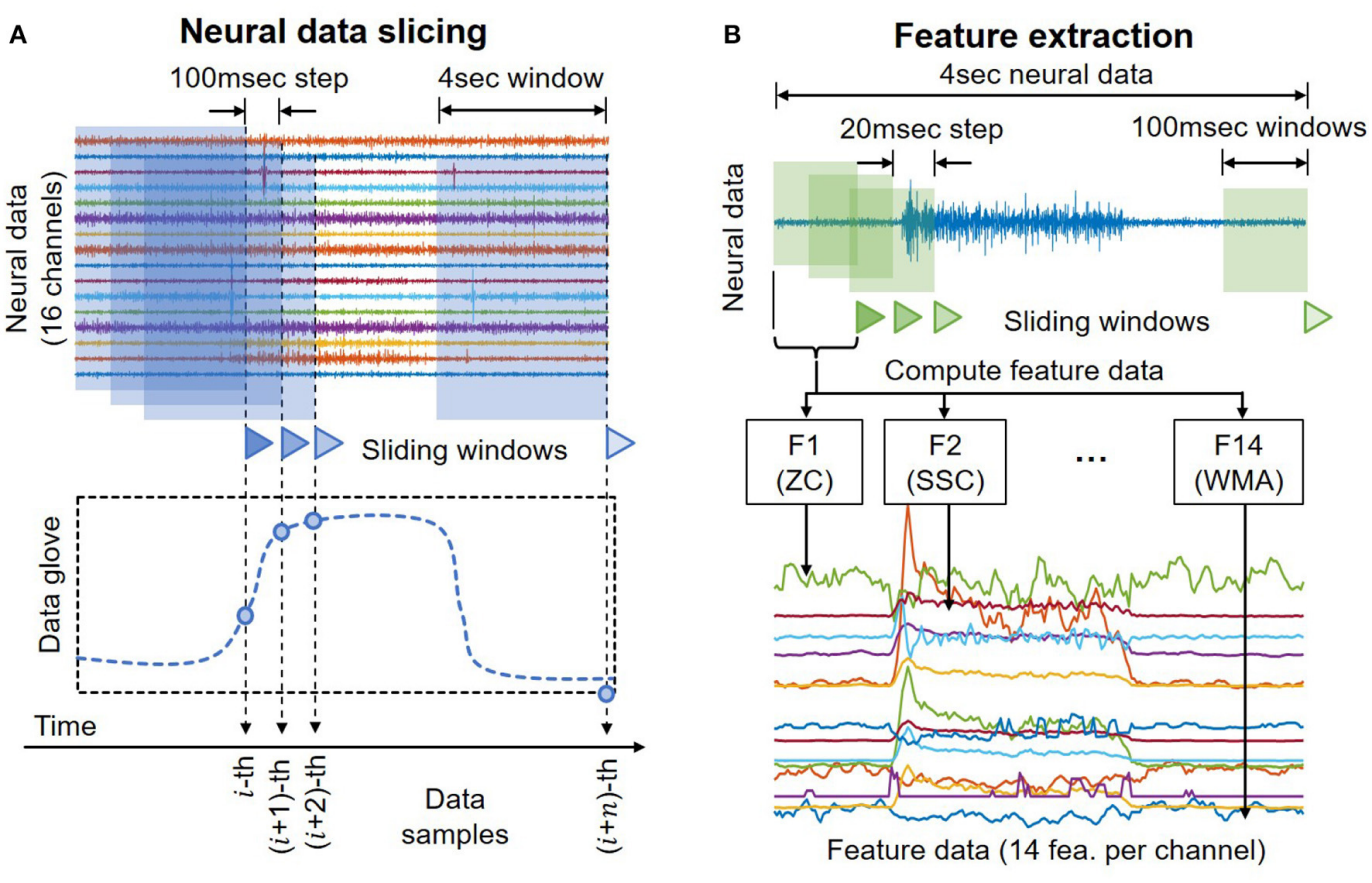
**(A)** Illustration of the sliding windows to cut neural data to create a pseudo-online dataset that resembles conditions in online decoding. **(B)** Illustration of the process to compute the feature data.

Raw neural data are cut using a sliding window to resemble online motor decoding (Figure 3A). Here the window's length is set to 4 s with an incremental step of 100 ms. At any instant of time, the decoder can only observe the past neural data. The pseudo-online dataset contains overlapping windows from a total of 50.7 min worth of neural recordings. Each of these 4 s neural data segments serves as an input trial of the motor decoding process later.

### 3.2. Feature Extraction

Previous studies have shown that feature extraction is an effective gateway to achieve optimal classification performance with signals in the low-frequency band by highlighting critical hidden information while rejecting unwanted noise and interference. Here we select 14 of the most simple and robust features that are frequently used in previous motor decoding studies (Zardoshti-Kermani, 1995; Phinyomark, 2009, 2012; Rafiee, 2011). They are chosen such that there is no linear relationship between any pair of features. All features can be computed in the temporal domain with relatively simple arithmetic, thus aiding the implementation in future portable systems.

**Figure 4:**
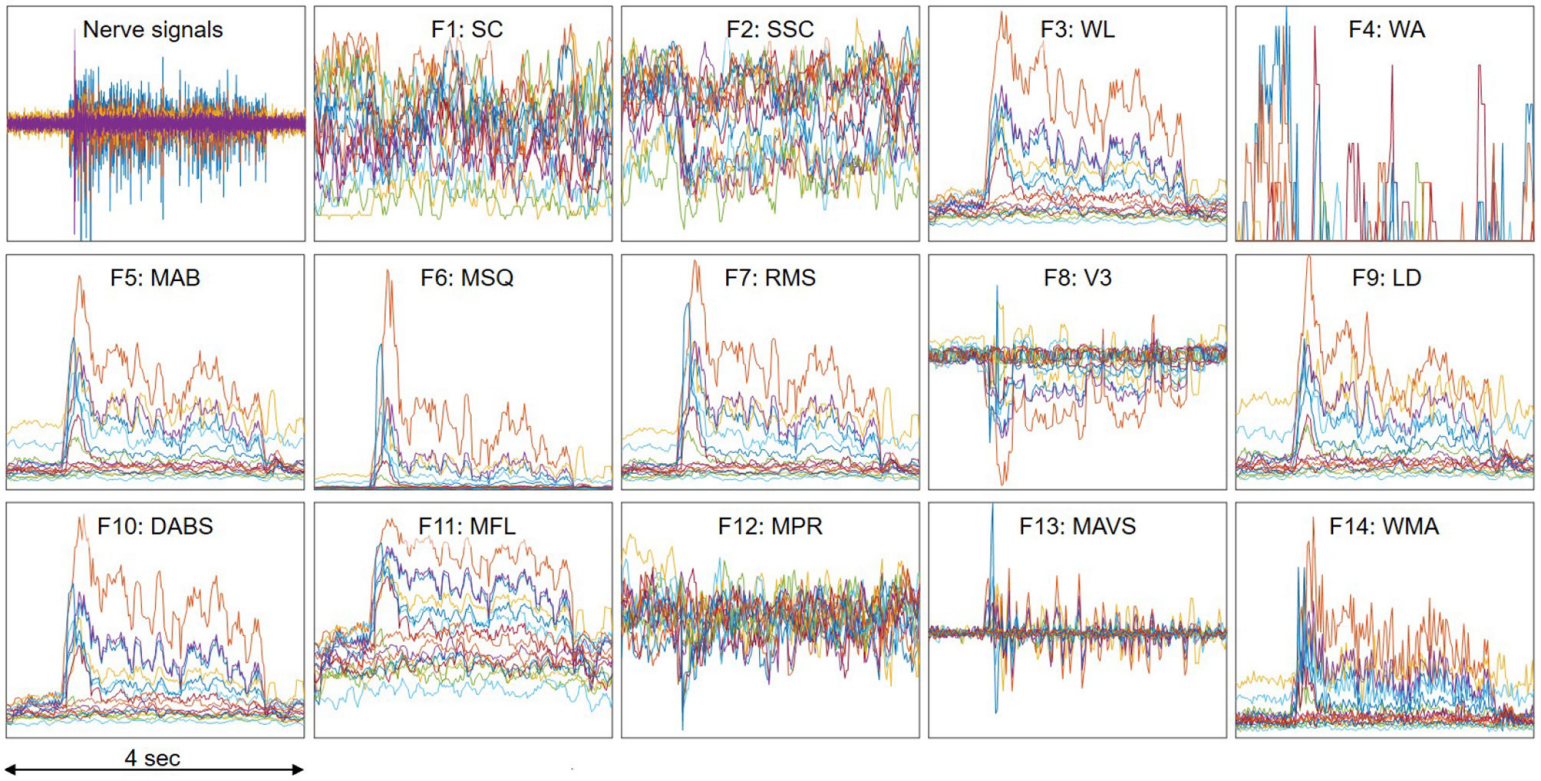
An example of feature data in one trial which shows clear correlation with the finger's movement. A trial includes the finger's movement from resting to fully flexing and back to resting. Each color represents one of the 16 recording channels. The amplitude of each feature is normalized by a fixed value.

Table 1 summaries the descriptions and formula of the features. Figure 3B illustrates the process to compute feature data. *x*_*i*_ is the 4 s neural data segments, which are further divided into windows of 100 ms with *N* is the window length. Two consecutive windows are 80% overlapped, which is equivalent to a 20 ms time step. This results in a data stream of 224 features over 16 channels with a data rate of 179.2 kbps (11.2 kbps per channel), which is more than 40 times lower than the raw data rate. Figure 4 presents an example of the feature data in one trial that shows a clear correlation between the changes of the 14 extracted features and the finger's movement. The amplitude of each feature is normalized by a fixed value before feeding to the deep learning models.

**Table 1:** List of features, descriptions, and formula.

	**Feature**	**Description**	**Formula**
F1	Zero crossing (ZC)	The number of times the demeaned data change sign.	$\Sigma_{i=2}^{N}\text{sgn}\left(-x_{i-1}x_{i}\right)$
F2	Slope sign changes (SSC)	The number of times the differential data change sign.	$\Sigma_{i=3}^{N}\text{sgn}\left[-\left(x_{i}-x_{i-1}\right)\left(x_{i-1}-x_{i-2}\right)\right]$
F3	Waveform length (WL)	The summation of the absolute values of the differential data.	$\Sigma_{i=2}^{N}|x_{i}-x_{i-1}|$
F4	Wilson amplitude (WA)	The number of times the change in the signal amplitudes of two consecutive samples exceeds the standard deviation.	$\Sigma_{i=2}^{N}\text{sgn}\left(|x_{i}-x_{i-1}|-x_{std}\right)$
F5	Mean absolute (MAB)	The average of the absolute values of the data.	$\frac{1}{N}\Sigma_{i=1}^{N}|x_{i}|$
F6	Mean square (MSQ)	The average of the square values of the data.	$\frac{1}{N}\Sigma_{i=1}^{N}x_{i}^{2}$
F7	Root mean square (RMS)	The root of MSQ or v-order 2.	$\sqrt{\frac{1}{N}\Sigma_{i=1}^{N}x_{i}^{2}}$
F8	V-order 3 (V3)	The cubic root of the average of the cube of the data.	$\sqrt[{3}]{\frac{1}{N}\Sigma_{i=1}^{N}x_{i}^{3}}$
F9	Log detector (LD)	The exponential of the average of the log data.	$\text{exp}\left(\frac{1}{N}\Sigma_{i=1}^{N}\text{log}|x_{i}|\right)$
F10	Difference absolute standard deviation (DABS)	Standard deviation of the absolute of the differential data.	$\sqrt{\frac{1}{N-1}\Sigma_{i=2}^{N}{\left(x_{i}-x_{i-1}\right)}^{2}}$
F11	Maximum fractal length (MFL)	Equivalent to the log of DABS minus an offset that is equal to 1/2log(*N*−1)	$\text{log}\left[\sqrt{\Sigma_{i=2}^{N}{\left(x_{i}-x_{i-1}\right)}^{2}}\right]$
F12	Myopulse percentage rate (MPR)	The number of times the absolute of the data exceeds the standard deviation.	$\Sigma_{i=1}^{N}\text{sgn}\left(|x_{i}|-x_{std}\right)$
F13	Mean absolute value slope (MAVS)	A modified version of MAV that is the difference between the MAV of the first half of a signal window and the second half.	$\frac{\Sigma_{i=1}^{\left\lfloor N/2\right\rfloor }|x_{i}|-\Sigma_{i=\left\lfloor N/2\right\rfloor +1}^{N}|x_{i}|}{\left\lfloor N/2\right\rfloor }$
F14	Weighted mean absolute (WMA)	A modified version of the MAB where the first and last 25% of a signal window is given less weight than the middle 50%.	$\frac{1}{N}\Sigma_{i=1}^{N}w_{i}|x_{i}|$ where *w*_*i*_ = 1 if *i*∈[0.25*N*, 0.75*N*], and *w*_*i*_ = 0.5 otherwise

## 4. Proposed Deep Learning Models and Decoding Strategies

### 4.1. Two-Step (2S) Strategy

Each finger exists in a binary state: active or inactive, depending on the patient's intent to move it or not. There are 32 different combinations of five fingers corresponding to 32 hand gestures. Only a few gestures are frequently used in daily living activities, such as bending a finger (“10000”, “01000”, …, “00001”), index pinching (“00011”), or grasp/fist (“11111”). Therefore, classifying the hand gesture before regressing the fingers' trajectories would significantly reduce the possible outcome and lead to more accurate predictions. The movement of inactive fingers could also be set to zero, which lessens the false positives when a finger “wiggles” while it is not supposed to.

**Figure 5:**
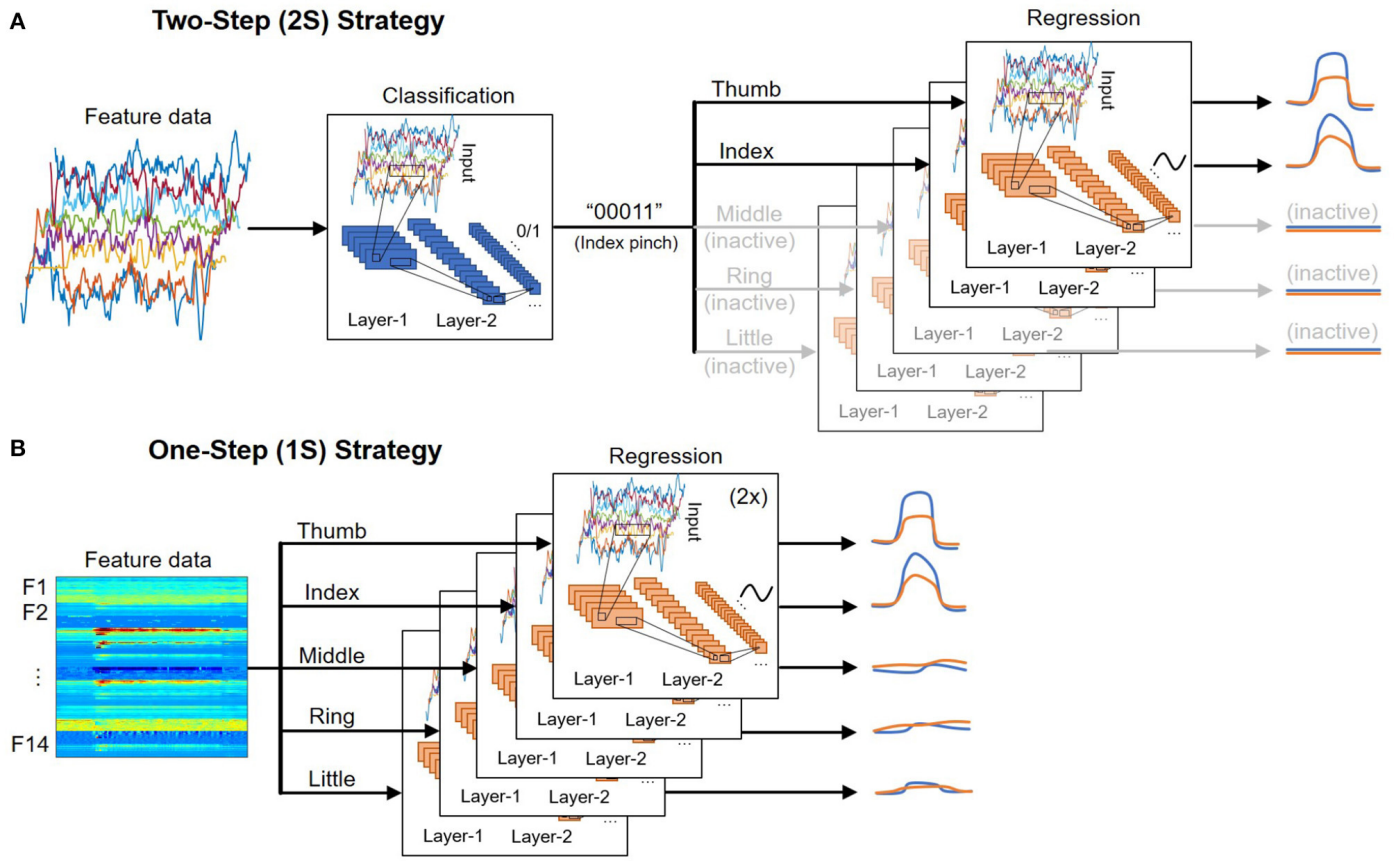
Illustration of the **(A)** two-step (2S) and **(B)** one-step (1S) strategy for deploying deep learning models.

Figure 5A shows an illustration of the 2S strategy. In the first step, the classification output is a [1 × 5] vector encoding the state of five fingers. While the dataset used in this study only includes nine possible outcomes, the system can be easily expanded in the future to cover more hand gestures by appending the dataset and fine-tuning the models. In contrast, many past studies focus on classifying a specific motion, which requires modifying the architecture and re-training the models to account for additional gestures.

In the second step, the trajectory of each DOF is regressed by a deep learning model. Ten separate models regress the trajectory of 10 DOF (two per finger). The models associated with inactive fingers are disabled, and the prediction outputs are set to zero. As a result, the dataset used to train each model is only a subset of the full dataset where the corresponding DOF is active. While all models use the same architecture, they are independently optimized using different sets of training parameters such as learning rate, minibatch size, number of epochs, etc., to achieve the best performance. An advantage of this approach is that if one DOF fails or has poor performance, it would not affect the performance of others.

### 4.2. One-Step (1S) Strategy

Figure 5B shows an illustration of the 1S strategy. It is the most straightforward approach where the trajectories of each DOF are directly regressed regardless of the fingers' state. As a result, the full dataset, which includes data when the DOF is active (positive samples) and idle (negative samples), must be used to train each DOF. Because the number of negative samples often exceeds the number of positive samples from 5:1 to 10:1, additional steps such as data augmentation and/or weight balancing need to be done during training. This also leads to more false-positives where an idle DOF still has small movements that could affect the overall accuracy.

Although the 1S procedure is more straightforward than the 2S's, its time-latency and efficiency are not necessarily better than the 2S. While our hands are at rest most of the time, the 1S approach has to continuously predict all fingers' trajectories regardless of their activeness status. The 2S approach goes through a classification step to identify the active DOF before predicting the trajectories of those DOF, which helps disable several deep learning models depending on the hand gestures, direct resources to the active DOF, and results in lower overall latency and computation in most implementations where there is only one processing unit (GPU or CPU) in the second step. It is shown in **Table 3** that a simple RF can help achieve comparable classification outcomes of accuracy above 0.99 to those from the two deep learning techniques. The regression steps of both approaches utilize the same deep learning model.

### 4.3. Deep Learning Models

**Figure 6:**
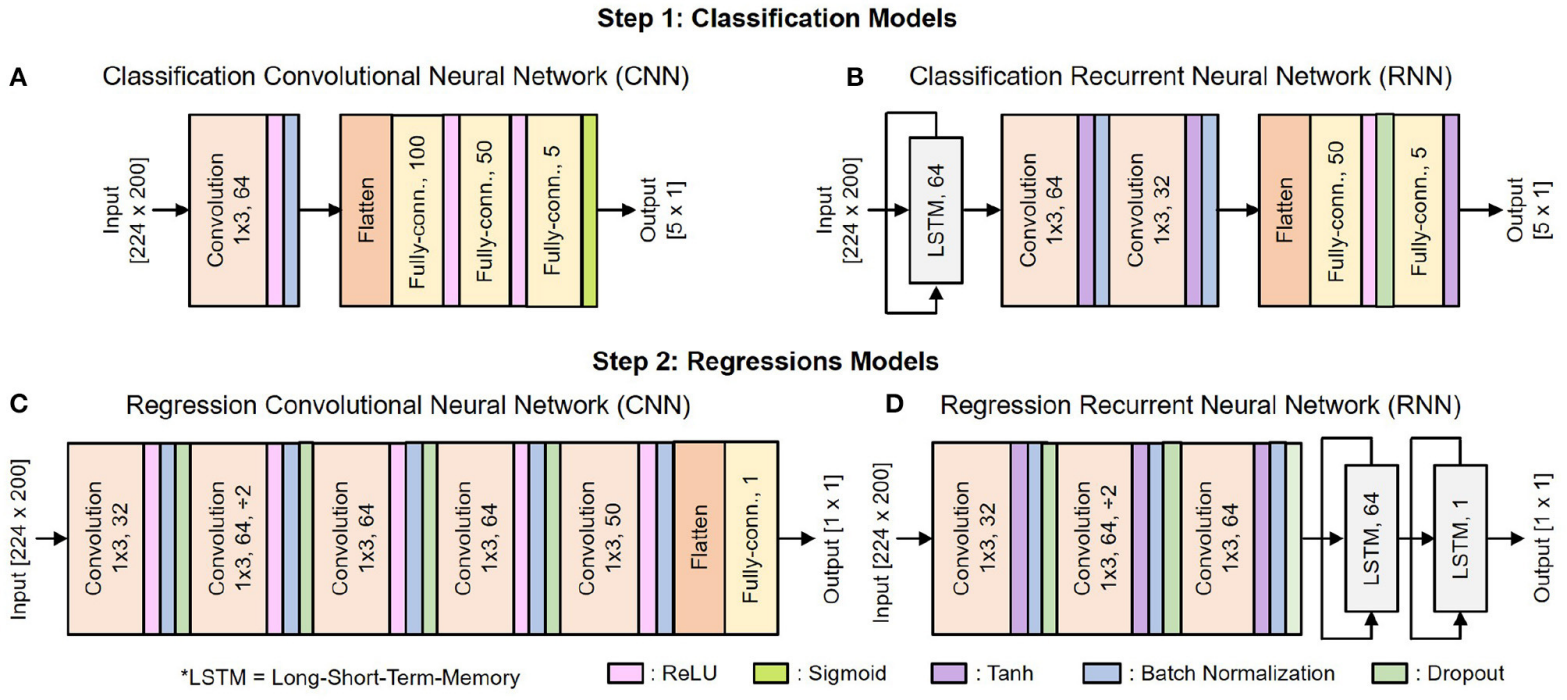
Architecture of the deep learning models: **(A)** CNN for classification, **(B)** RNN for classification, **(C)** CNN for regression, **(D)** RNN for regression.

Figure 6 shows the architecture of the deep learning classification and regression models. They include standard building blocks such as convolutional, long-short term memory (LSTM), fully-connected, and dropout layers of different combinations, order, and set of parameter values. The architecture is optimized by gradually adding layers and tuning their parameters while tracking the decoder's efficacy using 5-fold cross-validation. As the performance converges, additional layers would tend to result in over-fitting.

There are 10 copies of the regression model for 10 DOF, each of which is trained separately. We use Adam optimizer with the default parameters β_1_ = 0.99, β_2_ = 0.999, and a weight decay regularization $L_{2}=10^{-5}$. The mini-batch size is set to 38, with each training epoch consists of 10 mini-batches. The learning rate is initialized to 0.005 and reduced by a factor of 10 when the training loss stopped improving for two consecutive epochs.

As shown in Figure 6, the input of both 1S and 2S networks is a matrix [224 x 200] where 224 is the total number of features, and 200 is the time vector. During the data preprocessing step, raw neural data are cut into several 4 s segments. Each of these 4 s segments goes through the feature extraction step, during which it is further cut into 100 ms segments with an incremental step of 20 ms as illustrated in Figure 3B. There are 14 features to be extracted from each channel as mentioned in Table 1. Hence, a 4 s segment of raw neural data from 16 channels results in a matrix [224 × 200] where 224 is the total number of extracted features from 16 channels [16 × 14 = 224] and 200 is the total number of time steps cut from 4 s segments [4 s/20 ms = 200].

Table 2 shows a rough comparison between the deep learning models used in this and our previous work. Note that for regression, the number of learnable parameters is a total of 10 models for 10 different DOF. The addition of feature extraction, thus dimensional reduction, allows significantly lowing the deep learning models' size and complexity. This is essential for translating the proposed decoding paradigm into a real-time implementation for portable systems.

**Table 2:** Comparison between this work and Nguyen and Xu (2020).

	**No. of layers**			**No. of parameters**
	**Conv**.	**LSTM**	**Fully conn**.	
Nguyen and Xu (2020)	21	2	3	25,927,050
This work (classification)	1	0	3	1,465,749
This work (regression)	3	2	0	767,200

## 5. Experimental Setup

In this research, we investigate the performance of two main deep learning architectures: the CNN and RNN for both classification and regression tasks. Besides, the deep learning models are benchmarked against “classic” supervised machine learning techniques as the baseline. They include support vector machine (SVM), random forest (RF), and multi-layer perceptron (MLP).

For baseline techniques, the input of the classification task is the average of 224 features across 200 time-steps, while the input of the regression task is the 30 most important PCA components. The SVM models use the radial basis function (RBF) for classification and polynomial kernel of degree three for regression, with parameter *C* = 1. The RF models use 5 and 10 trees for classification and regression, respectively, with a max depth of three. The MLP model for classification is created by replacing the convolutional layer of the CNN model with a fully-connected layer of 200 units. The MLP model for regression has four layers with 300, 300, 300, and 50 units, respectively.

The 5-fold cross-validation is used to compare the performance of the classification task. For the regression task, the dataset is randomly split with 80% for training and 20% for validation. The split is done such that no data windows from the training set overlap with any data windows from the validation set.

The data processing is done in MATLAB (MathWorks, MA, USA). The deep learning networks are implemented in Python using the PyTorch ^2^ library. The deep learning models are trained and evaluated on a desktop computer with an Intel Core i7-8086K CPU and an NVIDIA TITAN Xp GPU.

## 6. Metrics and Results

### 6.1. Metrics

This subsection introduces the metrics to measure the performance of five models, including the two discussed deep learning models and three other supervised machine learning techniques as benchmarks in both classification and regression tasks.

The performance of the classification task is evaluated using standard metrics including accuracy and F1 score derived from true-positive (TP), true-negative (TN), false-positive (FP), and false-negative (FN) as follows:

(1) $$\begin{array}{ll} \text{Sensitivity} & =\text{TP}/\left(\text{TP}+\text{FN}\right) \end{array}$$

(2) $$\begin{array}{ll} \text{Specificity} & =\text{TN}/\left(\text{TN}+\text{FP}\right) \end{array}$$

(3) $$\begin{array}{ll} \text{Precision} & =\text{TP}/\left(\text{TP}+\text{FP}\right) \end{array}$$

(4) $$\begin{array}{ll} \text{Accuracy} & =\left(\text{Sensitivity}+\text{Specificity}\right)/2 \end{array}$$

(5) $$\begin{array}{ll} \text{F1 Score} & =\frac{2\cdot \text{Sensitivity}\cdot \text{Precision}}{\text{Sensitivity}+\text{Precision}} \end{array}$$

We use the definition of accuracy with an equal weight of sensitivity and specificity because the occurrence of class-1 (active finger) is largely outnumbered by the occurrence of class-0 (inactive finger).

The performance of the regression task is quantified by two metrics: mean squared error (MSE) and variance accounted for (VAF). MSE measures the absolute deviation of an estimated from the actual value of a DOF, while VAF reflects the relative deviation from the actual values of several DOF. They are defined as follows:

(6) $$\begin{array}{l} \text{MSE}\left(y,ŷ\right)=\frac{1}{N}\Sigma_{i=1}^{N}{\left(ŷi-yi\right)}^{2} \end{array}$$

(7) $$\begin{array}{l} \text{VAF}\left(y,ŷ\right)=1-\frac{\Sigma_{i=1}^{N}{\left(ŷi-yi\right)}^{2}}{\Sigma_{i=1}^{N}{\left(yi-ȳi\right)}^{2}} \end{array}$$

where *N* is the number of samples, *y* is the ground-truth trajectory, ȳ is the average of *y*, and ŷ is the estimated trajectory. The value of *y* and ŷ are normalized in a range [0, 1] in which 0 represents the resting position.

Although the MSE is the most common metric and effectively measures absolute prediction errors, it cannot reflect the relative importance of each DOF to the general movements. For example, if the average magnitude of DOF A is hundreds of times smaller than that of DOF B, a bad estimation of A still can yield lower MSE than a reasonable estimation of B. The VAF score is more robust in such scenarios; thus, it could be used to compare the performance between DOF of different magnitude. The value of the VAF score ranges from (-∞, 1], the higher, the better. This research presents the MSE and VAF scores of different neural decoding models from different approaches to choose the best one for future clinical applications in real-time. A neural decoder that results in negative VAF scores is definitely not the best one. In fact, SVM is the only decoding architecture that occasionally shows negative VAF scores. Therefore, ignoring its negative VAF scores would neither affect the comparison nor change the final decision of the best model. For practicality, negative VAF values are ignored when presenting the data.

### 6.2. Classification Results

Table 3 shows the average 5-fold cross-validation classification results of all the techniques. The predictability of each finger is largely different from one another. The thumb, which produces strong signals only on the median nerve (first eight channels), is easily recognized by all techniques. Overall, CNN offers the best performance with accuracy and an F1 score for all fingers exceeding 99%. RF closely follows with performance ranging from 98 to 99%. While deep learning still outperforms classic techniques, it is worth noting that RF could also be a prominent candidate for real-world implementation because RF can be more efficiently deployed in low-power, portable systems.

**Table 3:** Classification performance.

	**Accuracy**					**F1 score**				
	**Thumb**	**Index**	**Middle**	**Ring**	**Little**	**Thumb**	**Index**	**Middle**	**Ring**	**Little**
SVM	0.999	0.767	0.916	0.895	0.932	0.999	0.808	0.911	0.771	0.677
RF	0.999	0.975	0.996	0.992	0.988	0.999	0.976	0.996	0.980	0.981
MLP	0.999	0.965	0.973	0.966	0.970	0.999	0.965	0.972	0.954	0.945
CNN	0.999	0.999	0.999	0.999	0.999	0.999	0.999	0.999	0.999	0.999
RNN	0.999	0.948	0.988	0.970	0.994	0.999	0.957	0.987	0.950	0.994

### 6.3. Regression Results

**Figure 7:**
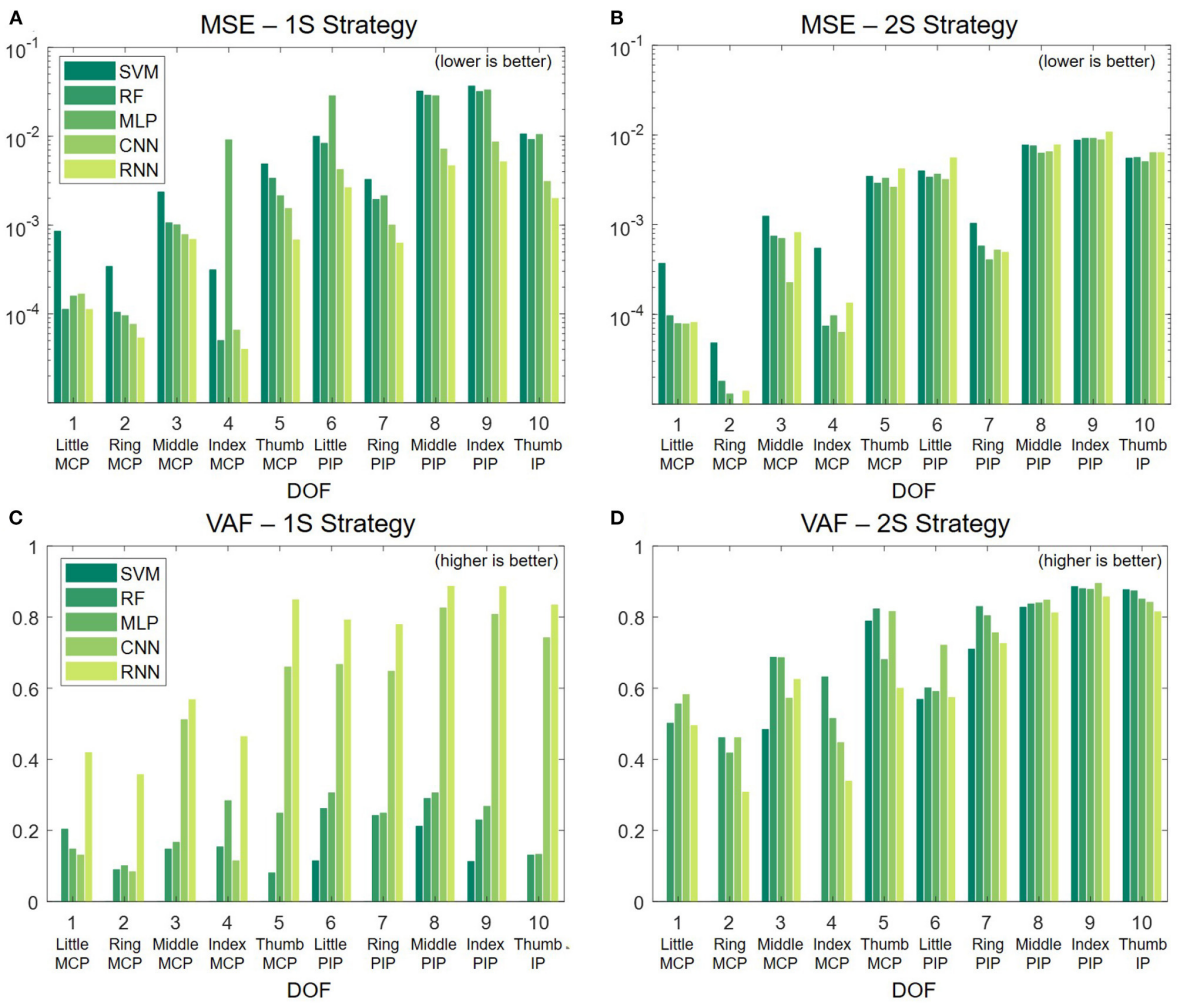
Regression performance in term of MSE **(A,B)** and VAF **(C,D)**.

Figure 7 presents the MSE and VAF scores for both strategies. In the 1S approach, the deep learning models, especially RNN, significantly outperform the other methods in both MSE and VAF, as shown in Figures 7A,C. To verify the significance of differences between RNN and the other decoders, we conduct paired t-tests with a Bonferroni correction. The results indicate that the performance differences in MSE and VAF are all statistically significant with *p* < 0.001. In the 2S approach, the performance is more consistent across all methods, where classic methods even outperform deep learning counterparts in certain DOF as shown in Figures 7B,D. Between the two strategies, the 1S approach generally gives better results; however, the high performance can only be achieved with RNN.

## 7. Discussion

### 7.1. Feature Extraction Reduces Decoders' Complexity

Both deep learning architectures investigated in this study, namely CNN and RNN, deliver comparable motor decoding performance to our previous work (Nguyen and Xu, 2020) while require much lower computational resources to implement. The average VAF score for most DOF is 0.7, with some exceeding 0.9. Such results are achieved with relatively shallow deep learning models with 4–5 layers, a significant reduction from the previous implementation with 26 layers. While an extra step of feature extraction is required, all feature extraction techniques are specifically designed to be efficiently computed with conventional arithmetic processors (e.g., CPU) and/or hardware accelerators (e.g., FPGA, ASIC).

Another direction for future works would be including additional features. Here we apply the most common features in the temporal domain for their simplicity and well-established standing for neuroprosthesis applications. There are other features, such as mean power, median frequency, peak frequency, etc., in the frequency domain that have been explored in previous literature. The purpose of this study is not to exhaustively investigate the effect of all existing features in motor decoding but to prove the effectiveness of the feature extraction method in achieving decent decoding outcomes. The success of this method will open up to future research on simultaneously applying more features in multiple domains for better outcomes.

Furthermore, it is worth noting that the use of feature extraction excludes most of the high-frequency band 600–3,000 Hz, which is shown in our previous work that could contain additional nerve information associated with neural spikes. A future direction would be extracting that information using spike detection and sorting techniques and combine them with the information of the low-frequency band to boost the prediction accuracy. However, the computational complexity must be carefully catered to not hinder the real-time aspect of the overall system.

### 7.2. Classic Machine Learning v.s. Deep Learning

The classification task can be accomplished with high accuracy using most classic machine learning techniques (e.g., RF) and near-perfect with deep learning approaches (e.g., CNN). Along with other evidence in Nguyen and Xu (2020), this suggests that nerve data captured by our neural interface contain apparent neural patterns that can be clearly recognized to control neuroprostheses. While the current dataset only covers 9/32 different hand gestures, these are still promising results that would support future developments, including expanding the dataset to cover additional gestures.

The regression results are consistent with the conclusion of many past studies that deep learning techniques only show clear advantages over classic machine learning methods when handling a large dataset. This is evident in the 1S strategy, where each DOF is trained with the full dataset consisting of all possible hand gestures. In contrast, in the 2S strategy, where the dataset is divided into smaller subsets, deep learning techniques lose their leverage. However, as the dataset is expanded in the future, we generally believe that deep learning techniques should emerge as the dominant approach.

### 7.3. Improving Motor Decoder Effectiveness

This paper focuses on exploring different approaches to build a new decoding paradigm that is simpler and requires less computational power than that of the previous research for applying in real-time applications. However, for such real-time applications, we also need to test the deep learning decoders' stability in the long run. Future research would apply the same deep learning architectures but train and validate on data recorded on different days. The time gap between the training and validation data may also be varied to investigate the trade-off between the re-training frequency and the decoding outcomes' accuracy.

## 8. Conclusion

This work presents several approaches to optimize the motor decoding paradigm that interprets the motor intent embedded in the peripheral nerve signals for controlling the prosthetic hand. The use of feature extraction largely reduces the data dimensionality while retaining essential neural information in the low-frequency band. This allows achieving similar decoding performance with deep learning architectures of much lower computational complexity. Two different strategies for deploying deep learning models, namely 2S and 1S, with a classification and a regression stage, are also investigated. The results indicate that CNN and RF can deliver high accuracy classification performance, while RNN gives better regression performance when trained on the full dataset with the 1S approach. The findings layout an important foundation for the next development, which is translating the proposed motor decoding paradigm to real-time applications, which requires not only accuracy but also efficiency.

## Data Availability Statement

Further information and requests for resources and reagents should be directed to and will be fulfilled by Zhi Yang (yang5029@umn.edu) and Qi Zhao (qzhao@umn.edu) in accordance with the IRB protocol.

## Ethics Statement

Written informed consent was obtained from the individual(s) for the publication of any potentially identifiable images or data included in this article.

## Author Contributions

ZY and EK conceptualized the system and experiments. DL, MJ, and QZ designed, trained and benchmarked the deep learning models. AN and JX designed the integrated circuits and carried out hardware integration. EK designed the microelectrode array. JC and EK performed the implantation surgery and post-operative care. DL, AN, JX, and MD conducted experiments, collected nerve data, and performed data analysis. DL and AN prepared the manuscript. All authors contributed to the article and approved the submitted version.

## Conflict of Interest

ZY is co-founder of, and holds equity in, Fasikl Inc., a sponsor of this project. This interest has been reviewed and managed by the University of Minnesota in accordance with its Conflict of Interest policy. JC and EK have ownership in Nerves Incorporated, a sponsor of this project. The remaining authors declare that this study received funding from Fasikl Inc. The funder had the following involvement with the study, including study design, data collection and analysis, and preparation of the manuscript.

## Footnotes

^1^https://clinicaltrials.gov/ct2/show/NCT02994160

^2^https://pytorch.org/
